# A Case of Postpartum Anogenital Mammary-Like Gland Tumor with Focal Lactational Features: A Nomenclature Issue

**DOI:** 10.1155/2019/6703248

**Published:** 2019-03-12

**Authors:** Sakinah A. Thiryayi, Marjan Rouzbahman, Danny Ghazarian

**Affiliations:** Department of Pathology, Laboratory Medicine Program, University Health Network and University of Toronto, 11th Floor Eaton Wing, Toronto General Hospital 200 Elizabeth Street, Toronto, Canada M5G 2C4

## Abstract

Mammary-like glands (MLG) are considered to be a normal constituent of the anogenital region and can give rise to tumors with variable morphology that may be difficult to classify. We present a case of an anogenital mammary-like gland tumor in a breastfeeding woman showing morphological variation with lactational change, an unusual finding. We discuss the differing terminology used to report these tumors and the variation in assignment of their origin to MLG or ectopic breast tissue.

## 1. Introduction

The term anogenital mammary-like glands (MLG) was proposed by van der Putte who also elucidated them as a normal anogenital region constituent occurring at the junction of the skin and cloaca-derived mucosa which, in females, results in their location primarily along the interlabial sulcus and inner side of the labia majora [1]. These glands vary from slightly coiled simple wide tubular structures to ones with multiple branches and acini forming lobules. The glandular lining comprises basal myoepithelial cells and luminal columnar epithelium with ‘snouts' that becomes cuboidal in the acini. MLG show luminal shedding of epithelial clusters, as well as expression of estrogen and progesterone receptors. The immunohistochemical profile of MLG has recently been shown to be similar to that of normal breast tissue [2].

Whilst the term anogenital mammary-like glands has gained acceptance, there is not universal agreement on the origin of these glands. van der Putte argued against the traditional theory of supernumerary mammary tissue originating from the mammary ridges and noted the ultrastructural presence of electron lucent secretory granules in MLG, a finding not present in mammary epithelium. The presence of both ectopic breast tissue and MLG in the anogenital region has also been proposed [3]. We describe a case of MLG tumor showing secretory change in a breastfeeding woman.

## 2. Case Report

A 36-year-old woman developed a painless cyst on the inner right labium majus at approximately 6 months of gestation which remained stable during the rest of the pregnancy. She delivered by cesarean section and the cyst was excised two months later, at which time she was breastfeeding.

**Figure 1:**
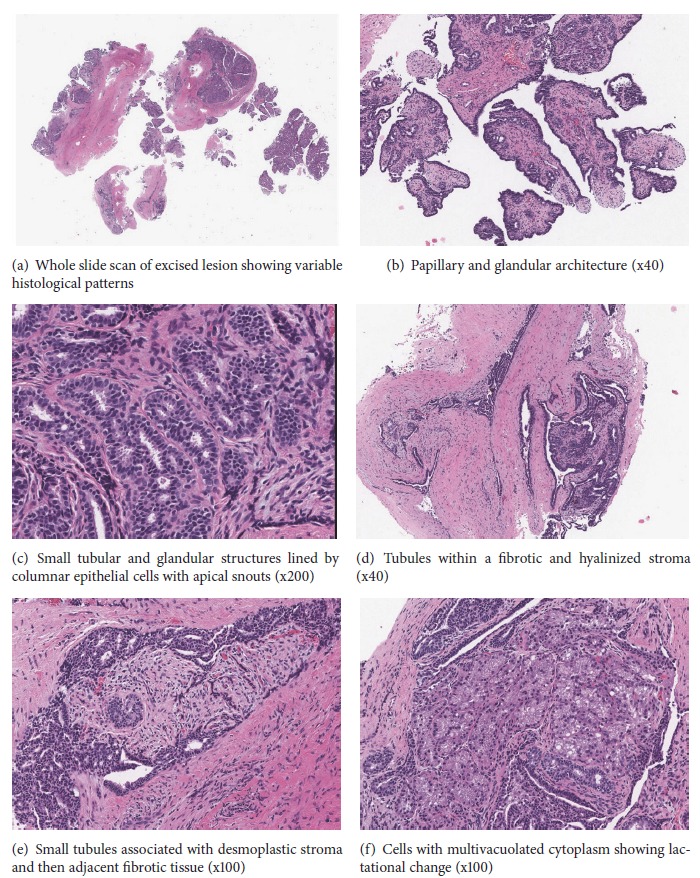


The excision comprised multiple irregular pieces of pale tan tissue up to 1.3cm in aggregate. Microscopic examination showed a disrupted tumor with morphologically variable but intermingled areas (Figure 1(a)). Much of the tumor demonstrated hidradenoma papilliferum type architecture with papillary and glandular areas in which the surface/luminal lining cells were bland cuboidal to low columnar with round to oval hyperchromatic nuclei, absent nucleoli, and scant eosinophilic cytoplasm (Figure 1(b)). Apical snouts were evident in areas (Figure 1(c)). Elsewhere closely packed small tubules were associated with desmoplastic and hyalinized stroma (Figures 1(d) and 1(e)). Other glandular spaces showed cells with abundant pale multivacuolated cytoplasm similar to mammary lactational change (Figure 1(f)). The features were those of a mammary-like gland tumor showing hidradenoma papilliferum-like areas, tubular adenoma features, and lactational change. No definite nonneoplastic mammary-like glands were noted in the peritumoral tissue.

**Figure 2:**
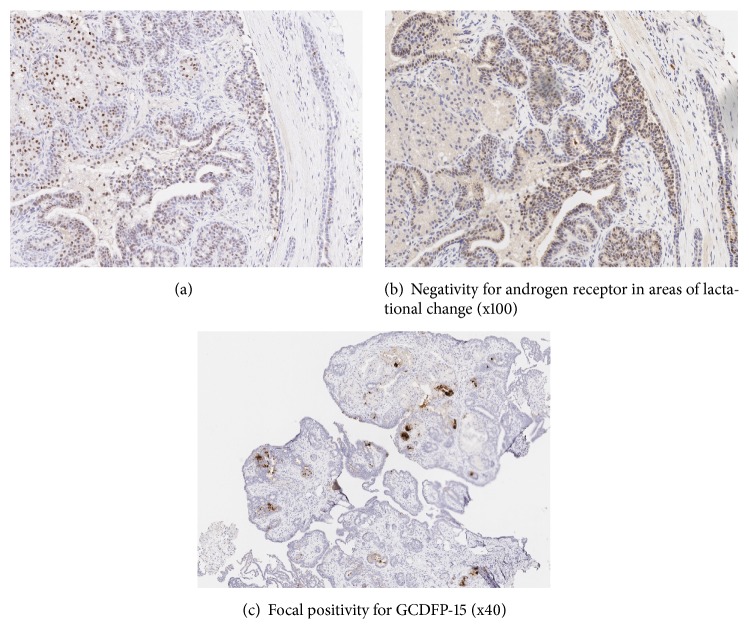


Immunohistochemistry showed diffuse positivity for CK7, CK18, and GATA3, the latter two weaker in the lactational areas. There was patchy positivity for estrogen receptor (ER) throughout the lesion (Figure 2(a)) whilst androgen receptor (AR) showed patchy staining in the nonlactational areas (Figure 2(b)). Very occasional cells were positive for progesterone receptor (PR) and there was focal staining for GCDFP-15 (Figure 2(c)). Calponin and p63 highlighted the basal myoepithelial cells. There was variable staining for MIB-1 with foci of increased activity which generally corresponded to CK5 positive areas that had an elongated ductal or tubular morphology.

## 3. Discussion

While there remain divergent theories on the origin of mammary like tissue in the vulva, the nomenclature of lesions arising from MLG has also not been standardized. Kazakov et al. [4] reviewed the range of lesions related to MLG, correlating them with mammary counterparts with several epithelial and stromal changes showing identical histological appearances at both sites. The commonest benign neoplasm affecting MLG is hidradenoma papilliferum (HP). Scurry et al. [5] reviewed 46 cases which they stated are more accurately termed MLG adenoma. A large study of 264 HP documented the histopathological changes in these tumors, emphasizing mammary-type alterations and their proximity to anogenital MLG, but did not refer to them as MLG adenomas [6].

Other reports have used various terms to refer to lesions related to MLG (Table 1) with some cases, the present one included, showing mixed features which do not neatly fit into the previously recognized entities in this region or their counterparts in the breast. The concept of MLG hyperplasia is not well described.

The morphological similarity of anogenital MLG lesions and their breast counterparts has been found to extend to the genetic level too with molecular assessment detecting similar gene mutations involving the PI3K-AKT cascade present in tumors at both sites. Genes coding for various kinases were the site of most mutations [12].

The fragmented nature of the specimen in our case, as well as the variable morphological appearances which did not entirely correspond to those previously described in HP, made this lesion difficult to categorize. An interesting element was the presence of lactational type secretory change. A previous case of HP in pregnancy [13] raised the possibility that pregnancy-related hormonal alterations could affect the histologic appearance of HP. In contrast to our case, this case showed moderate positivity for PR whilst staining for ER was negative. Both retention and lack of expression of hormone receptors have been documented in various breast neoplasms during pregnancy with 25 lactating adenomas noted to be negative for ER, PR, and AR whilst the lactating lobules surrounding infiltrating ductal carcinoma showed weak (1-5%) positivity for these markers [14]. Lactational change adds another element to be included in the nomenclature of these lesions. While there are case reports describing lactational change in fibroadenomas within vulvar ectopic breast tissue in breastfeeding women [15, 16], a case of fibroadenoma with lactation-like change involving anogenital MLG [17] serves to highlight the differing interpretations of mammary like tissue in this region. The hormonal changes associated with pregnancy and breastfeeding have been noted to result in growth of such lesions.

In order to facilitate communication and avoid long cumbersome descriptive diagnoses, we propose that the spectrum of tumors recognized as MLG adenoma be expanded beyond those that would have been called hidradenoma papilliferum to include these other histologically variable lesions, some of which may not have a clear correlate in the breast.

## Figures and Tables

**Table 1:** Diagnostic terminology and histological features used to describe lesions arising from anogenital mammary-like glands.

Study	Diagnostic term	Descriptive features
Scurry et al. [5]	Mammary-like gland adenoma	Diverse histological appearances, combinations of cystic and solid areas, and tubular and papillary growth. Apocrine metaplasia common (57%). Constituent stroma sclerotic or desmoplastic, variable in amount.

Donati et al. [7]	Adenoma of anogenital mammary-like glands	Abundant fibrous stroma surrounding lobular arrangements of glands associated with branching ducts.

Ahmed et al. [8]	Adenoma of anogenital mammary-like glands	Glands and ducts within a dense fibrotic stroma reminiscent of breast intracanalicular fibroadenoma.

Konstantinova et al. [9]	Composite neoplastic lesion of the vulva with mixed features of fibroadenoma and hidradenoma papilliferum combined with pseudoangiomatous stromal hyperplasia	Branching, elongated glands within a cellular stroma, and anastomosing tubules separated by fibrous tissue and focal papillae.

Nishie et al. [10]	Hidradenoma papilliferum with mixed histopathologic features of syringocystadenoma papilliferum and anogenital mammary-like glands	Exo- and endophytic papillary tumor with maze-like pattern, abundant fibrous tissue and apocrine epithelium. In the deep dermis were underlying large tubular glands with surrounding fibrous tissue resembling normal mammary glands.

Kazakov et al. [11]	Unusual hyperplasia of anogenital mammary-like glands	Complex compact lobular proliferation of small ducts with fibroadenomatous and adenosis like areas.
